# Cost-Effectiveness of Oral Immunotherapy Treatments vs No Treatment for Peanut Allergy in Children

**DOI:** 10.1001/jamanetworkopen.2026.2410

**Published:** 2026-03-20

**Authors:** Li Huang, Melanie Lloyd, Adam Franz, Paxton Loke, Michael O’Sullivan, Michael Gold, Patrick Quinn, Mimi L. K. Tang, Kim Dalziel

**Affiliations:** 1 Child Health Economics Unit, The University of Melbourne, Victoria, Australia; 2 Allergy Immunology, Murdoch Children’s Research Institute, Victoria, Australia; 3 Health Economics and Policy Evaluation Research, Monash Institute of Pharmaceutical Sciences, Victoria, Australia; 4 Department of Paediatrics, The University of Melbourne, Victoria, Australia; 5 Immunology Department, Perth Children’s Hospital, Western Australia, Australia; 6 Division of Paediatrics, The University of Western Australia, Western Australia, Australia; 7 The Kids Research Institute Australia, Western Australia, Australia; 8 Department of Allergy and Immunology, Women’s and Children’s Hospital, South Australia, Australia; 9 Discipline of Paediatrics, Adelaide University, South Australia, Australia; 10 Department of Allergy and Immunology, The Royal Children’s Hospital, Victoria, Australia

## Abstract

**Importance:**

The first peanut oral immunotherapy (OIT) for children was approved by the US Food and Drug Administration (FDA) in 2020. While clinical efficacy is established, evidence on cost-effectiveness—whether the benefits outweigh the costs and adverse effects—remains limited. A variant of OIT, known as probiotic and peanut OIT (PPOIT), has shown similar efficacy in trials.

**Objective:**

To compare the cost-effectiveness of PPOIT, OIT, and no treatment.

**Design, Setting, and Participants:**

This economic evaluation was conducted alongside a multicenter, randomized, placebo-controlled clinical trial in Australia between 2016 and 2019. Time horizon was 10 years, including 1.5 years of active treatment, 2 years of posttreatment follow-up, and 6.5 years of extrapolation. Data were analyzed from May 2024 to August 2025.

**Interventions:**

PPOIT and OIT.

**Main Outcomes and Measures:**

Effectiveness was measured using remission achieved and patient quality-adjusted life years (QALYs) gained. Costs were evaluated from a health care payer perspective, including active treatment and adverse event costs, and were calculated in Australian dollars. Incremental cost-effectiveness ratios were estimated. Sensitivity analyses were conducted to capture uncertainty.

**Results:**

A total of 201 children aged 1 to 10 years were recruited, 79 in PPOIT, 83 in OIT, and 39 in no treatment (mean [SD] age, 5.9 [2.8] years; 129 [64.2%] male). Over a 10-year horizon, mean (SD) cost per patient was A$3956 (A$67) for PPOIT (treatment, A$3579 [A$0]; adverse events, A$377 [A$67]), A$3582 [A$57] for OIT (treatment, A$3179 [A$0]; adverse events, A$402 [A$57]), and A$249 (A$87) for no treatment (treatment, A$0 [A$0]; adverse events, A$249 [A$87]). Mean (SD) annual remission was 34.1% (12.7%) for PPOIT, 35.1% (15.4%) for OIT, and 7.3% (8.1%) for no treatment. The total QALYs gained for PPOIIT, OIT, and no treatment were 0.096, 0.055, and 0, respectively. PPOIT was slightly more costly than OIT, achieved similar remission, and achieved better quality-of-life. Compared with no treatment, both treatments were more costly with minor risks, achieved higher remission (PPOIT, A$1384; 95% CI, A$1269-A$1415; and OIT, A$1199; 95% CI, A$1091-A$1217 per year of remission achieved, respectively), and improved quality of life (PPOIT, A$38 435; 95% CI, A$31 058-A$48 668 and OIT, A$60 840; 95% CI, A$49 479-A$86 531 per QALY gained, respectively).

**Conclusions and Relevance:**

This economic evaluation found that for remission, both PPOIT and OIT were cost-effective and good value compared with no treatment, with OIT associated with a larger effect size but no clinically meaningful difference. When QALYs are prioritized, PPOIT offers the best value. Key factors associated with cost-effectiveness were treatment product pricing and patient quality of life.

## Key Points

**Question:**

Are oral immunotherapy (OIT) treatments cost-effective for managing peanut allergy in children?

**Findings:**

This economic evaluation conducted alongside a clinical trial involving 201 children found that both probiotic peanut OIT (PPOIT) and peanut OIT were cost-effective compared with no treatment when remission was the effectiveness outcome. When effectiveness was assessed using quality-adjusted life years, PPOIT offered the best value.

**Meaning:**

These findings suggest that PPOIT and OIT present good value compared with no treatment for achieving remission.

## Teaser

This economic evaluation compares the cost-effectiveness of peanut oral immunotherapy and probiotic peanut oral immunotherapy with no treatment among children with peanut allergy in Australia.

## Introduction

Peanut allergy affects 2% to 3% of children in high-income countries.^1,2^ Management has historically relied on strict avoidance. Oral immunotherapy (OIT) is an emerging treatment option that can desensitize a considerable proportion of patients through gradually increasing doses of peanut protein, with a subset achieving clinical remission (sustained unresponsiveness).^3^ Desensitization refers to a temporary increase in reaction threshold, while remission refers to the absence of clinical reactivity after discontinuing treatment for a period of time, although relapse may occur. In 2020, the US Food and Drug Administration (FDA) approved Palforzia (Stallergenes Greer), the first peanut OIT for children. Palforzia desensitizes 67% of patients to 600mg of peanut protein (roughly 2 peanuts), and is indicated to mitigate allergic reactions from accidental peanut exposure.^4^

Despite FDA approval of Palforzia for its clinical effectiveness, its cost-effectiveness, and the cost-effectiveness of peanut OIT more broadly, remains uncertain; that is, whether OIT’s benefits outweigh its costs and adverse effects. The primary concerns are the cost of treatment and maintenance dosing, and the uncertain effects of treatment-related adverse events on patient quality-of-life.^5^ Nevertheless, in 2022, the UK National Institute for Health and Care Excellence (NICE) officially recommended Palforzia as a treatment option—not standard of care—for peanut allergy in children and young people, believing that its cost-effectiveness is likely acceptable for use of national resources.^6^ In Australia, the Australasian Society of Clinical Immunology and Allergy currently states that, while some patients with food allergy may benefit from OIT, strict avoidance remains the recommended standard of care for most patients.^7^

Probiotic and peanut OIT (PPOIT), a combination of a probiotic adjuvant with OIT, has demonstrated similar efficacy to standalone OIT with slightly reduced gastrointestinal adverse events in trials.^8,9,10,11^ Two existing publications (trial-based comparison of PPOIT vs placebo^12^ and model-based comparison of PPOIT vs no treatment^13^) concluded that PPOIT is cost-effective. Currently, no cost-effectiveness evidence is available directly comparing PPOIT with OIT. This study addresses that gap by evaluating the cost-effectiveness of PPOIT compared with the same OIT regimen without probiotic, and to no treatment, alongside the PPOIT-003 trial. We also contribute to the broader evidence base on the cost-effectiveness of peanut OIT.

## Methods

### The PPOIT-003 Trial

PPOIT-003 was a multicenter, randomized, placebo-controlled, Phase 2b trial conducted at 3 Australian tertiary hospitals between 2016 and 2019, followed by 2 years of follow-up extending to the end of 2021, in children aged 1 to 10 years when recruited with peanut allergy confirmed by double-blind placebo-controlled food challenge (DBPCFC).^11,14^ Children were randomized to 18 months of treatment with PPOIT, OIT, or placebo; 176 completed the trial (88%). Treatment involved (1) a 1-day rush phase where participants received a single dose of probiotic or placebo and increasing doses of peanut protein or placebo every 20 minutes from 0.1 mg to 12 mg; (2) a build-up phase beginning the following day with a fixed daily dose of probiotic or placebo taken together with a daily dose of peanut protein or placebo increasing every 2 weeks from 25 mg up to 2000 mg over 16 weeks; and (3) a maintenance phase of a single dose of probiotic or placebo and 2000 mg of peanut protein or placebo daily until completing a total of 18 months of treatment. At the end of treatment (approximately 1.5 years), participants underwent a DBPCFC to assess desensitization (cumulative 5g peanut protein) and those who passed proceeded to a second DBPCFC 8 weeks later to assess remission (cumulative 5g peanut protein), with strict peanut elimination in between.

The primary trial outcome, remission, was induced in 46% of PPOIT-treated, 51% of OIT-treated, and 5% of placebo-treated participants at the end of treatment.^10^ Depending on outcome (remission, desensitization, or allergic), patients were advised to eat peanut ad libitum, continue a daily treatment of 1 to 2 peanuts, or avoid peanut, respectively. A total of 151 of 176 eligible (86%) enrolled in trial follow-up.^11^ Trial design, outcomes and 2-year follow-up results were reported previously.^10,11^ Adverse events and patient quality-of-life were collected via parent surveys. The intention-to-treat (ITT) population was used in this study.

This economic evaluation was approved by the human research ethics committee at the Royal Children’s Hospital Melbourne. Parents or guardians provided written informed consent. Analyses were reported following the Consolidated Health Economic Evaluation Reporting Standards (CHEERS) reporting guideline.^15^

### Statistical Analysis

Costs were evaluated from a health care payer perspective and included the costs of active treatment and adverse events. Effectiveness was assessed using remission achieved and patient quality-adjusted life years (QALYs) gained. A time horizon of 10 years was used, including 1.5 years of treatment delivery, 2 years of posttreatment follow-up, and 6.5 years of extrapolation thereafter.

Cost of active treatment was estimated from the treatment protocol and trial records using a microcosting approach and included treatment preparation, rush phase, build-up phase, maintenance phase, and food challenge assessments. Staff-to-patient ratios for dosing supervision were adjusted to reflect clinical practice rather than a research trial setting (eTable 1 in Supplement 1). For example, during the rush phase, it was assumed that 1 nurse would supervise 3 patients simultaneously in practice, with 1 physician nearby on-call. Publicly available wage rates and on-costs were used to cost staff time (eTables 2 and 3 in Supplement 1). Pathology tests and medical and pharmaceutical supplies, including the PPOIT and OIT products were costed using sourced unit costs (eTable 2 in Supplement 1). Two food challenges were costed: 1 to assess desensitization and a subsequent 1 to assess remission in applicable patients.

Treatment costs for those who enrolled but did not complete treatment (25 children) were also included, assuming they finished half the maintenance phase. These costs were shared among completers in the reported cost per patient. Costs for matched placebos were not included for pragmatic relevance of results. For the placebo group, medical staff time providing care, dose supervision, and food challenges was costed to reflect that the enhanced medical care could impact quality-of-life.

Costs of adverse events that were either treatment-related or likely treatment-related were included. Those unlikely to be related, such as elective surgical procedures and head injuries, were excluded. Adverse events requiring emergency care such as ambulance calls, emergency department visits, and hospitalizations were costed using applicable unit costs (eTable 4 in Supplement 1). Minor and mild adverse events that did not require escalation of care were costed based on typical use of over the counter medications (eTable 4 in Supplement 1).

Remission during follow-up was assumed if patients reported continuing consumption of peanuts at 1-year and 2-year follow-ups, respectively. To estimate QALYs, generic, preference-weighted quality-of-life scores were required—allowing comparisons across conditions for societal resource allocation—multiplied by the corresponding duration of life. As generic quality-of-life was not available, condition-specific Food Allergy Quality of Life Questionnaire-Parent Form (FAQLQ-PF)^16^ collected at baseline, end of treatment, 1-year, and 2-year follow-ups were mapped to the generic Assessment of Quality-of-life-6D (AQoL-6D) utility values using a published Australian algorithm.^17^ In this study, quality of life refers to the mapped AQoL-6D scores, generally ranging from 0 to 1, with 1 representing full health and 0 representing dead.

For patients with missing outcomes, we assumed data were likely missing at random based on previous investigations.^10^ A random sampling from the observed outcome distributions within treatment groups with 1000 replications was used to preserve uncertainty with results presented in probabilistic sensitivity analysis.

Extrapolation beyond the trial follow-up was conducted because remission and quality-of-life gains from the active treatments are unlikely to disappear immediately once follow-up ends. A pessimistic and parsimonious 6.5-year extrapolation period was used, assuming that benefits diminish gradually over time, with further differences beyond assumed inconsequential.

Extrapolation is based on the following assumptions: (1) remission rates for both the PPOIT and OIT groups would decline following the trend observed between the final 2 follow-up points, estimated as the mean of the 2 given the minimal differences observed; (2) the remission rate for the placebo group would increase—contrary to the observed trend—to 10% by year 10;^18^ and (3) quality of life in all 3 groups would return to their respective baselines by year 10 based on general adaptation.^19,20^ For both remission and quality-of-life, variations around the means at the last available follow-up were used in extrapolation to preserve uncertainty (eFigure 1 in Supplement 1). Health care costs for adverse events during extrapolation were assumed to follow the same patterns and distributions observed during follow-up within each group.

While placebo treatment is essential in the trial to estimate remission gains, it has limited applicability for clinical implementation. A hypothetical no treatment scenario was constructed to approximate the standard care of peanut avoidance. For no treatment, costs of active treatment were assumed to be A$0 (all amounts in 2022 Australian dollars unless otherwise indicated with alternative currency signs; £1 is equal to A$1.5 Australian and $1 US is equal to A$1.4 Australian, based on the 2022 Purchasing Power Parity published by the Organization for Economic Cooperation and Development Statistics), while adverse event costs and remission outcomes were drawn from the placebo group. Quality of life was assumed to neither increase nor decrease in the absence of additional medical care provided by the trial; that is, it remains constant on average.

The cost-effectiveness of PPOIT compared with OIT was assessed using the incremental cost-effectiveness ratio (ICER), defined as the difference in cost divided by the difference in effectiveness. When remission was the effectiveness outcome, the ICER was interpreted as the cost per additional year of remission achieved. When QALY was the effectiveness outcome, the ICER was interpreted as the cost per additional QALY gained. The same method was used to compare PPOIT vs no treatment, and OIT vs no treatment. In the base case analysis, cost-effectiveness is assessed using a value judgment threshold of A$5000 per year of remission achieved (caregivers were on average willing to pay approximately US A$3504 per year per child for food allergy treatment based on a US research in 2011),^21^ and A$50 000 per QALY gained.

To account for sampling uncertainty, probabilistic sensitivity analysis was performed using simulation drawing from the observed distributions for costs and outcomes, and the results are presented using CIs and acceptability curves. One-way sensitivity analysis was used for the following scenarios: (1) using the NICE-listed OIT price, with PPOIT costed as OIT plus the probiotic component; (2) applying a 3.5% annual discount rate to costs and outcomes beyond the treatment period; (3) assuming remission rates observed at the 2-year follow-up are sustained over the remaining time horizon for PPOIT and OIT; and (4) assuming quality-of-life outcomes observed at 2-year follow-up are sustained for PPOIT and OIT over the remaining time horizon. Finally, a threshold analysis was conducted to assess the range of OIT prices at which treatments would be cost-effective compared with no treatment based on QALY thresholds from Australia, the UK, and the US. The Stata statistical software package version 14.0 (StataCorp) was used and analyses were conducted from May 2024 to August 2025.

## Results

**Figure 1.**
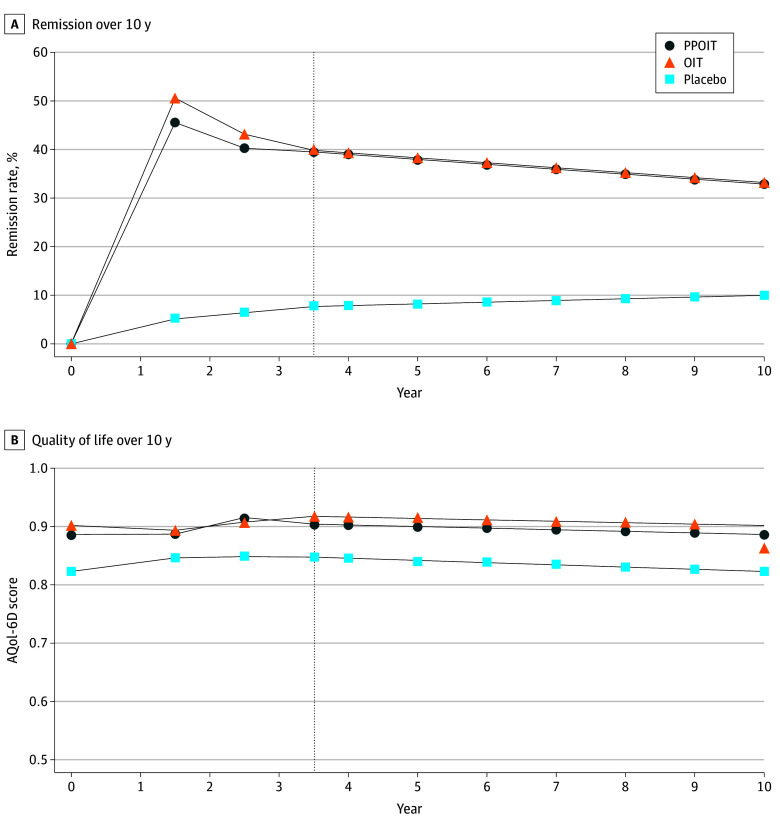
Line Graph Showing Remission and Quality-of-Life Outcomes Over the 10-Year Time Horizon AQoL-6D indicates Assessment of Quality-of-life-6D (scores generally range from 0 to 1, with higher scores indicating better health); OIT, peanut oral immunotherapy; PPOIT, probiotic and peanut oral immunotherapy.

A total of 201 children were included: 79 in PPOIT, 83 in OIT, and 39 in the placebo group (mean [SD] age, 5.9 [2.8] years; 129 [64.2%] male). Remission rates and quality-of-life scores at end of treatment and during follow-ups for each group are reported in Table 1. Quality-of-life scores were similar to baseline at end-of-treatment in the PPOIT group, declined in the OIT group, and improved in the placebo group (Figure 1).

**Table 1.** Patient Characteristics, Remission Rates, and Quality-of-Life Outcomes From the Trial and 1- and 2-Year Follow-Ups

Variable	Mean (SD)		
	PPOIT (n = 79)	OIT (n = 83)	Placebo (n = 39)
Age at trial entry, y	6.0 (3.0)	5.8 (2.7)	6.0 (2.7)
Sex, No. (%)			
Female	30 (38.0)	28 (33.7)	14 (35.9)
Male	49 (62.0)	55 (66.3)	25 (64.1)
Remission rate, No. %			
At end of treatment	36 (45.6)	42 (50.6)	2 (5.1)
1-y posttreatment follow-up^a^	32 (40.5)	36 (43.4)	2.5 (6.4)^b^
2-y posttreatment follow-up^a^	31 (39.2)	33 (39.8)	3 (7.7)^b^
AQoL-6D score^c^			
Baseline	0.886 (0.103)	0.902 (0.112)	0.823 (0.138)
At end of treatment	0.887 (0.114)	0.894 (0.124)	0.846 (0.134)
1-y posttreatment follow-up	0.915 (0.095)	0.908 (0.100)	0.845 (0.126)
2-y posttreatment follow-up	0.901 (0.086)	0.920 (0.123)	0.838 (0.136)

Abbreviations: AQoL-6D, Assessment of Quality-of-life-6D (scores generally range from 0 to 1 with higher scores indicating better health); PPOIT, probiotic and peanut oral immunotherapy; OIT, peanut oral immunotherapy.

^a^ Remission was not confirmed by double-blind, placebo-controlled food challenge during follow-ups. It was assumed that remission was maintained if patients reported continuing peanut consumption at 1-year and 2-year follow-ups, respectively.

^b^ Three participants in the placebo group reported continued peanut consumption at the 2-year posttreatment follow-up, resulting in an estimated remission rate of 3 of 39 participants (7.7%). The 1-year posttreatment remission rate for the placebo group was estimated using linear interpolation between the end of treatment and the 2-year follow-up.

^c^ The total sample sizes for AQoL-6D in the 3 treatment groups were 200 at baseline, 178 at trial completion, 172 at the 1-year follow-up, and 118 at the 2-year follow-up.

Total cost per patient over 10 years was estimated to be A$3956 for PPOIT and A$3582 for OIT, with A$3579 (90%) and A$3179 (89%), respectively, attributable to treatment and the remaining for adverse events (Table 2). Most of the treatment cost was attributable to physician and nurse time (A$2174 [61%] for PPOIT and A$2138 [67%] for OIT), followed by the cost of the PPOIT and OIT products (A$1202 [34%] and A$845 [27%], respectively), and the remaining for pathology tests and epinephrine autoinjectors. Adverse events costs were higher for the 2 intervention groups compared with placebo. Estimated medical care costs were lower in the placebo group, primarily because few participants underwent remission testing. In terms of effectiveness, patients in the 2 intervention groups had higher remission rates. Total QALY gains were highest in the placebo group, followed by PPOIT, then OIT.

**Table 2.** Cost and Effectiveness Outcomes for a Time Horizon of 10 Years

Outcome	PPOIT	OIT	Placebo	No treatment
Cost per patient, A$				
Total	3956	3582	2381	249
Cost of active treatment	3579	3179	2132	0
Physicians or nurses	2174	2138	1982	0
PPOIT or OIT product	1202	845	0	0
Pathology tests and epinephrine autoinjectors	203	196	150	0
Cost of adverse events	377	402	249	249^a^
Active treatment phase (1.5 y)	308	285	222	222^a^
Emergency care (ambulance, ED, hospitalization)	95	60	155	155^a^
Nonemergency care (over-the-counter medications)	213	225	68	68^a^
Posttreatment follow-up phase (2 y)	16	28	6	6^a^
Post follow-up extrapolation phase (6.5 y)	53^b^	90^b^	20^b^	20^a^^,^^b^
Effectiveness per patient				
Mean annual remission rate, %	34.1 (12.7)	35.1 (15.4)	7.3 (8.1)	7.3 (8.1)^a^
End of treatment (at y 1.5)	45.6	50.6	5.1	5.1^a^
Posttreatment follow-up (at y 3.5)	39.5	39.8	7.7	7.7^a^
Post follow-up extrapolation (at y 10)	32.9^b^	33.2^b^	10.0^b^	10.0^a^^,^^b^
Total QALYs gained	0.096	0.055	0.147	0
During treatment (1.5 y)	0.001	−0.006	0.018	0
Posttreatment follow-up (2 y)	0.038	0.010	0.049	0
Post follow-up extrapolation (6.5 y)	0.058^b^	0.051^b^	0.080^b^	0

Abbreviations: A$, Australian dollars; ED, emergency department; PPOIT, probiotic and peanut oral immunotherapy; OIT, peanut oral immunotherapy; QALY, quality-adjusted life years.

^a^ Indicates adverse event costs and remission outcomes sourced from the placebo group.

^b^ Indicates extrapolation results.

The cost-effectiveness results are presented in Table 3, and all reported numerical estimates are statistically significant. This largely reflects the clear clinical benefits of active treatments on remission and the minimal variability in treatment costs, which dominate total costs (for cost and effectiveness differences, see eTable 5 in Supplement 1). Our interpretation therefore focuses on whether the magnitude of these differences is economically or clinically meaningful. Overall, PPOIT is slightly more costly than OIT primarily due to the probiotic component (mean cost difference, A$374; 95% CI, A$369-A$378) and has a slightly lower remission rate (mean annual remission difference, −1%; 95% CI, −2% to −0.1%), and thus is technically dominated in terms of remission; however, the differences are minimal and unlikely to be practically meaningful. When QALYs were the effectiveness outcome, PPOIT resulted in greater quality-of-life compared with OIT at a cost of A$8985 per additional QALY gained, well below conventional thresholds, and thus presents better value.

**Table 3.** Incremental Cost-Effectiveness Ratios (ICERs) Over a 10-Year Time Horizon

Cost	Base case	One-way sensitivity analysis			
		NICE OIT price	3.5% Discount rate		Remission sustained
Cost per y of remission gained, A$					
PPOIT vs OIT	OIT cheaper and better but difference minimal^a^	OIT cheaper and better but difference minimal^a^	OIT cheaper and better but difference minimal^a^		OIT cheaper and better but difference minimal^a^
PPOIT vs no treatment	1384 (1269-1415)^b^	4082 (3737-4209)^b^	1538 (1394-1584)^b^		1281 (1168-1299)^b^
OIT vs no treatment	1199 (1091-1217)^b^	3740 (3389-3815)^b^	1329 (1197-1363)^b^		1112 (1022-1135)^b^
Cost per QALY gained, A$					
PPOIT vs OIT	8985 (5120-17 592)^b^	12 872 (8455-19 774)^b^	9437 (6989-14 556)^b^		7778 (4572-13 377)^b^
PPOIT vs no treatment	38 435 (31 058-48 668)^b^	113 375 (91 315-142 357)	42 938 (36 832-48 749)^b^		24 069 (20 926-27 655)^b^
OIT vs no treatment	60 840 (49 479-86 531)	189 834 (154 668-270 540)	71 227 (57 413-83 078)		31 475 (29 010-38 416)^b^

Abbreviations: A$, Australian dollars; NICE, UK National Institute for Health and Care Excellence; PPOIT, probiotic and peanut oral immunotherapy; OIT, peanut oral immunotherapy; QALY, quality-adjusted life years.

^a^ It is not appropriate to report an ICER for dominant results (ie, cheaper and better) or when the differences are not economically or clinical meaningful.

^b^ ICER indicates good value (costing <A$5000 per additional year of remission achieved, or <A$50 000 per additional QALY gained).

**Figure 2.**
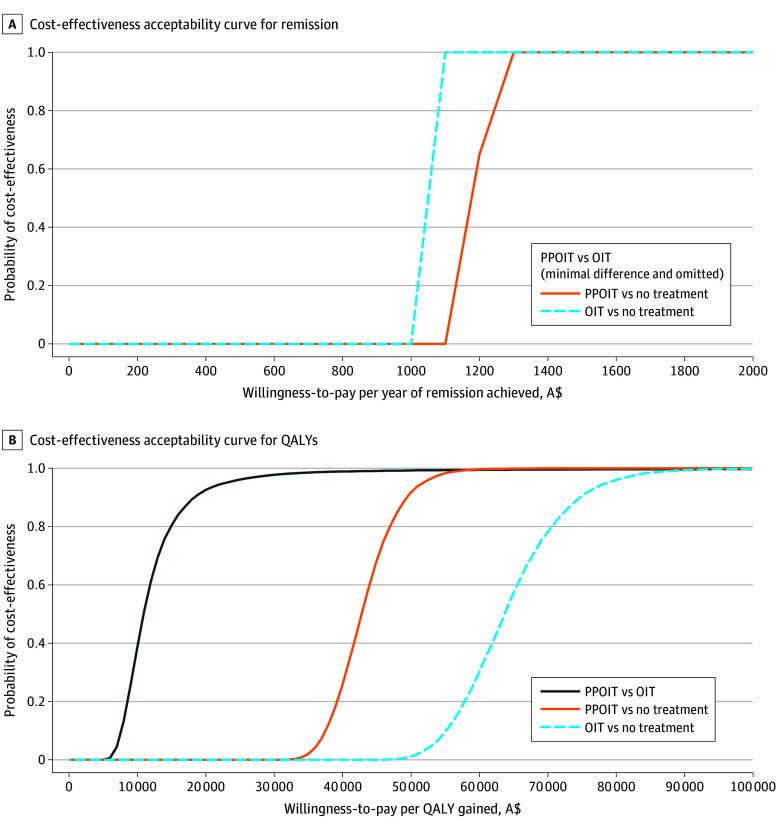
Cost-Effectiveness Acceptability Curves A$ indicates Australian dollars; OIT, peanut oral immunotherapy; PPOIT, probiotic and peanut oral immunotherapy; QALY, quality-adjusted life years.

Compared with hypothetical no treatment, both PPOIT and OIT were more costly, achieved higher remission, and improved quality-of-life. Both treatments are likely cost-effective in terms of remission, and PPOIT is also cost-effective in terms of QALYs. Probabilistic sensitivity analysis demonstrated confidence in these findings, with steep changes in acceptability curves associated with statistical significance and narrow CIs (Figure 2A). In terms of QALYs, PPOIT was cost-effective compared with both OIT and no treatment, with a greater than 90% probability of being good value at a willingness-to-pay of A$50 000 per additional QALY gained (Figure 2B).

One-way sensitivity analysis suggests that when the NICE-listed OIT price was applied (A$16 per day or A$8760 over 1.5 years) instead of the estimated cost in the base case (A$845 over 1.5 years), both active treatments became substantially more expensive and were no longer cost-effective for QALY gains compared with no treatment (Table 3). When sustained remission and quality-of-life outcomes are assumed rather than the pessimistic base case, the treatments were more cost-effective compared with no treatment. Threshold analysis is presented in eFigure 2 in Supplement 1, and within-trial follow-up results without extrapolation are presented in eTable 6 in Supplement 1. Threshold analysis indicated that PPOIT would be cost-effective for QALY outcomes at monthly OIT component prices of $22-$105 in the UK, below A$133 in Australia, and $16-$178 in the US.

## Discussion

PPOIT was slightly more expensive than OIT primarily due to the probiotic component; however, the total cost difference of A$374 over a 10-year time horizon is not considered economically meaningful in a high-income setting. OIT achieved slightly higher remission rates than PPOIT (<1% mean annual difference). Therefore, OIT is technically both less costly and more effective when remission is concerned; however, the differences are small and not practically meaningful. On the other hand, PPOIT achieved better quality-of-life compared with OIT, with an estimated cost of A$8985 per additional QALY gained (£5990 or US $6417 per QALY gained). This is well below the commonly referenced thresholds (£20 000-£30 000 and US $50 000-$100 000). Overall, the cost-effectiveness conclusion depends on what outcome is valued: if remission is valued, both active treatments are good values compared with hypothetical no treatment with OIT being slightly better; however, if QALYs are valued, PPOIT presents better value. The improved quality-of-life associated with PPOIT may be attributed to fewer gastrointestinal events.^10^ Although no difference in adverse event costs was observed, likely due to the minimal impact of gastrointestinal events on health care costs, these events may have a noticeable effect on quality-of-life.

When the NICE-listed Palforzia price was applied—approximately A$468 per month for the peanut component vs the trial-estimated A$50 per month, substantially higher treatment costs were estimated. While the treatments remain cost-effective considering remission compared with no treatment, costs per QALY gained were beyond the commonly accepted Australia threshold. Threshold analysis indicated that PPOIT would be cost-effective for QALY outcomes at monthly OIT component prices of $22-$105 in the UK, below A$133 in Australia, and $16-$178 in the US. While the NICE listed price may differ from the actual cost to the UK National Health Service due to confidential commercial agreements, the cost-effectiveness results remain sensitive to product pricing.

A positive placebo effect was observed, with the placebo group exhibiting higher quality of life than the treatment groups despite a low remission rate. However, caution is warranted in interpreting these findings. While placebo treatments may produce genuine benefits in certain settings,^22,23^ implementing an honest placebo treatment for peanut allergy is not practical; therefore, our hypothetical no treatment group better approximates standard avoidance care. The positive placebo effect on quality-of-life may stem from the increased care participants received from the trial, as well as the trial design, which allocated more patients to active treatments (79 in PPOIT and 83 in OIT) compared with placebo (39 patients)—this likely increased patients’ belief that they received active treatments while having few additional adverse events. Nevertheless, the placebo results remain informative, suggesting that patients value access to treatments and care, especially when associated with minimal additional adverse events.

### Limitations

Several limitations were identified. QALYs were estimated by mapping the condition-specific FAQLQ-PF to the generic AQoL-6D. The algorithm assigns greater weight to specific domains and severity levels based on population preferences.^24^ Future studies collecting generic quality of life directly would be highly valuable. Extrapolation was used to capture the longer-term outcomes beyond follow-up, as differences between groups were unlikely to converge immediately after follow-up ended. The extrapolation assumptions were pessimistic and may underestimate the true treatment outcomes. Food challenge screening was used in the trial to determine eligibility, but its cost was not included due to the difficulty appropriately capturing the benefits of delabeling allergy. However, a simple calculation suggests that if 10% of patients were excluded by food challenge as occurred in the trial, the cost savings from avoiding treatment alone would offset the cost of screening all patients. Adverse event costs may appear low because all groups received enhanced care during the trial, further underestimating the benefits of active treatments in a clinical setting. The current economic evaluation was conducted from a health care perspective. Further research that incorporates patient allergy- and treatment-related burdens, such as the condition burden associated with fear of high-risk allergic events in the absence of available treatments and the family cost of attending in-person treatment sessions, would be valuable.

## Conclusions

This economic evaluation found that PPOIT and OIT are similarly cost-effective for remission outcomes with OIT performing slightly better, whereas PPOIT is more cost-effective when considering QALY outcomes. The cost-effectiveness conclusions depend on the outcome of concern, indicating the importance of shared decision-making between patients and clinicians.

## Notes

Supplement 1. eTable 1. Number of Participants From the Trial and Medical Staff Ratio Assumed for Each Phase eTable 2. Unit Cost eTable 3. Medical Staff Time for Steps and Phases, and Other Medical Supplies eTable 4. Unit Cost for Adverse Events eTable 5. Difference in Key Cost and Effectiveness Outcomes eTable 6. Within Trial Follow-Up Cost-Effectiveness Results With no Extrapolation (3.5-Year Time Horizon) eFigure 1. Remission and Quality of Life eFigure 2 Threshold Analysis for OIT Price Per Month (Australian $) at Which the Treatments Would Be Cost-Effective Given QALY Thresholds for Australia, the UK and the US

Supplement 2. Data Sharing Statement
