# Supplementary material for: Cost-Effectiveness of Oral Immunotherapy Treatments vs No Treatment for Peanut Allergy in Children
Source: JAMA Netw Open. 2026 Mar 20;9(3):e262410. doi: 10.1001/jamanetworkopen.2026.2410 (PMC13005161; doi:10.1001/jamanetworkopen.2026.2410)
Supplement: Supplement 1. — eTable 1. Number of Participants From the Trial and Medical Staff Ratio Assumed for Each Phase eTable 2. Unit Cost eTable 3. Medical Staff Time for Steps and Phases, and Other Medical Supplies eTable 4. Unit Cost for Adverse Events eTable 5. Difference in Key Cost and Effectiveness Outcomes eTable 6. Within Trial Follow-Up Cost-Effectiveness Results With no Extrapolation (3.5-Year Time Horizon) eFigure 1. Remission and Quality of Life eFigure 2 Threshold Analysis for OIT Price Per Month (Australian $) at Which the Treatments Would Be Cost-Effective Given QALY Thresholds for Australia, the UK and the US [file jamanetwopen-e262410-s001.pdf]

Supplemental Online Content

Huang L, Lloyd M, Franz A, et al. Cost-effectiveness of probiotic peanut OIT, OIT, and no treatment for peanut allergy in children. *JAMA Netw Open*. 2026;9(3):e262410. doi:10.1001/jamanetworkopen.2026.2410

eTable 1. Number of Participants From the Trial and Medical Staff Ratio Assumed for Each Phase
eTable 2. Unit Cost
eTable 3. Medical Staff Time for Steps and Phases, and Other Medical Supplies
eTable 4. Unit Cost for Adverse Events
eTable 5. Difference in Key Cost and Effectiveness Outcomes
eTable 6 Within Trial Follow-Up Cost-Effectiveness Results With no Extrapolation (3.5-Year Time Horizon)
eFigure 1. Remission and Quality of Life
eFigure 2 Threshold Analysis for OIT Price Per Month (Australian $) at Which the Treatments Would Be Cost-Effective Given QALY Thresholds for Australia, the UK and the US

This supplemental material has been provided by the authors to give readers additional information about their work.

© 2026 Huang L et al. *JAMA Network Open*.

eTable 1. Number of Participants From the Trial and Medical Staff Ratio Assumed for Each Phase

	PPOIT	OIT	Placebo	Staff ratio*
**Screening**				
Patients screened, number	88	92	39	
Did not participate	9	9	0	
Patients enrolled, number	79	83	39	
Doctor ratio during preparation				1:1
Nurse ratio during preparation				1:3
**Rush phase, 1 visit**				
Doctor to patient ratio				1:3
Nurse to patient ratio				1:3
**Build up phase of 4 months, 8 visits**				
Doctor to patient ratio				1:5
Nurse to patient ratio				1:3
**Maintenance phase of 14 months, 5 visits**				
Doctor to patient ratio				1:5
Nurse to patient ratio				1:1
**End of trial food challenge**				
Dropout prior to food challenge	8	13	4	
Proceed to 1st food challenge	71	70	35	
Proceed to 2nd food challenge	61	61	2	
Remission	36	42	2	
Doctor ratio during food challenge				1:3
Nurse ratio during food challenge				1:3

*Nurse versus patient versus doctor ratios during rush and build up phase in the trial were 1:1:1 (i.e., 1 nurse in the same room with 1 patient; 1 doctor available in the vicinity on-call). In practice, ratios of 1:3:1 are anticipated when research tasks are excluded (i.e., 1 nurse in the same room with 3 patients; 1 doctor available in the vicinity on-call). The ratios applied are considered conservative given that the primary tasks for medical staff were to supervise dosing for safety purposes.

© 2026 Huang L et al. *JAMA Network Open*.

eTable 2. Unit Cost

	Unit cost	Data source
**Staff cost**		
Doctor, hourly	$125.1	Medical Specialists Enterprise Agreement, Year 1 specialist
Nurse, hourly	$37.6	Public Sector Nurses/Midwives Enterprise Agreement, Year 3 nurse
Doctor and nurse overhead	25%	Royal Children's Hospital
**Medical supply and care**		
PPOIT product, per two-week	$30	Probiotic and peanut powder. Probiotic price was assumed based on the over-the-counter price of for children (probiotic $10 per two-week; $20 per bottle with 30 capsules). Peanut powder price was assumed to be $20 per two-week based on the WHO Model List of Essential Medicines[a] ($1 per daily dose), applying a 40% monopoly premium[b] thus approximately $1.4 per daily dose.
Adrenaline autoinjector, two per pack	$158.78	Pharmaceutical Benefits Scheme (PBS) Australia. We assumed that 50% of patients still need a pack at screening despite they should have already.
Pathology initiation	$2.4	Pharmaceutical Benefits Scheme (PBS) Australia
Skin prick test	$40.05	Medicare Benefits Schedule (MBS) Australia, item 12003
Blood test, peanut sIgE	$26.8	Medicare Benefits Schedule (MBS) Australia, item 71079

[a]See "Hill AM, Barber MJ, Gotham D. Estimated costs of production and potential prices for the WHO Essential Medicines List. BMJ Global Health. 2018;3(1):e000571" for reference.
[b]See "High Generic Drug Prices and Market Competition. Annals of Internal Medicine. 2017;167(3):145-151" for reference.

© 2026 Huang L et al. *JAMA Network Open*.

eTable 3. Medical Staff Time for Steps and Phases, and Other Medical Supplies

Steps	Description	For each visit, patient receiving	Quantity unit
Treatment preparation	Doctor time (anaphylaxis action plan)	5	Min
	Nurse time (introducing treatment)	1	Hour
	Epipen Jr (two per unit)[a]	1	Unit
Rush phase, 1 visit per patient	Doctor time	4	Hour
	Nurse time	4	Hour
Buildup phase, 8 visits per patient	Doctor time	2.5	Hour
	Nurse time	2.5	Hour
Maintenance phase, 5 visits per patient	Doctor time	1	Hour
	Nurse time	1	Hour
Food challenge for allergic or desensitized	Doctor time	0.5	Day
	Nurse time	0.5	Day
	Pathology (skin prick and blood test)	1	Unit
Food challenge for remission	Doctor time	0.5	Day
	Nurse time	0.5	Day
	Pathology (skin prick and blood test)	1	Unit

Note: 1 day=7.25hours

[a]Assuming 50% of enrolled patients may still need despite they should have already.

© 2026 Huang L et al. *JAMA Network Open*.

eTable 4. Unit Cost for Adverse Events. NHCDC: National Hospital Cost Data Collection

	Unit cost ($)	Source
**Hospital inpatient episode**		
Asthma	1711	NHCDC, round 23
Dental surgery under general anaesthetic	1628	NHCDC, round 23
Infections	1514	NHCDC, round 23
Bone and joint injuries	1303	NHCDC, round 23
Abdominal Pain	1166	NHCDC, round 23
**ED visit**		
Admitted stay	965	NHCDC, round 22
Non-admitted stay	472	NHCDC, round 22
**Ambulance**		
Treatment without transport	546	Ambulance Victoria fee schedule 2019-20
**Out of hospital medical care**		
GP	38.75	Medicare Benefits Schedule Book
**Out of hospital pharmaceutical care**		
Aerius antihistamine	14.99	Over-the-counter price
Antibiotics	17.8	Over-the-counter price
Cetirizine	12.99	Over-the-counter price
Chlorsig	7.5	Over-the-counter price
Claratyne	10.99	Over-the-counter price
EpiPen (adrenaline autoinjector)	158.78	Pharmaceutical Benefits Scheme (PBS)
Erythromycin	20.16	Over-the-counter price
FESS	10.49	Over-the-counter price
Flixotide	19.49	Over-the-counter price
Hydrocortisone acetate	17.3	Over-the-counter price
Ibuprofen	22.99	Over-the-counter price
Montelukast	18.81	Over-the-counter price
Nasonex	15.49	Over-the-counter price
Panadol	18.49	Over-the-counter price
Phenergen	11.49	Over-the-counter price
Prednisolone	19.28	Over-the-counter price
Rhinocort	18.99	Over-the-counter price
Salbutamol	33.38	Over-the-counter price
Ventolin plus spacer	33.38	Over-the-counter price
Zyrtec	12.99	Over-the-counter price

Note: The cost of a full medication package was included regardless of the actual dose used. Australia local market price used.

© 2026 Huang L et al. *JAMA Network Open*.

eTable 5. Difference in Key Cost and Effectiveness Outcomes

PPOIT versus OIT	PPOIT	OIT	Difference (CI)
Cost	A$3956	A$3582	A$374 (A$369-A$378)
QALY gained	0.096	0.055	0.042 (0.019, 0.070)
Average remission rate per year	34.1%	35.1%	-1.0% (-2%, -0.1%)
PPOIT versus No treatment	PPOIT	No treatment	Difference (CI)
Cost	A$3956	A$249	A$3707 (A$3702, A$3713)
QALY gained	0.096	0	0.096 (0.076, 0.120)
Average remission rate per year	34.1%	7.3%	26.8% (26.2%, 29.5%)
OIT versus No treatment	OIT	No treatment	Difference (CI)
Cost	A$3582	A$249	A$3333 (A$3328, A$3337)
QALY gained	0.055	0	0.055 (0.037, 0.067)
Average remission rate per year	35.1%	7.3%	27.8% (27.5%, 30.3%)

eTable 6 Within Trial Follow-Up Cost-Effectiveness Results With no Extrapolation (3.5-Year Time Horizon)

Cost per year of remission gained	Base case (10-year time horizon)	Within trial follow up (3.5-year time horizon, no extrapolation)
PPOIT vs OIT	OIT cheaper & better but difference minimal[a]	OIT cheaper & better but difference minimal[a]
PPOIT vs No treatment	A$1,384 (A$1,269, A$1,415)	A$3,664 (A$3,627, A$3,706)
OIT vs No treatment	A$1,199 (A$1,091, A$1,217)	A$2976 (A$2,946, A$3,006)
**Cost per QALY gained**	**Base case (10-year time horizon)**	**Within trial follow up (3.5-year time horizon, no extrapolation)**
PPOIT vs OIT	A$8,985 (A$5,120, A$17,592)	A$11,684 (A$10631, A$12538)
PPOIT vs No treatment	A$38,435 (A$31,058, A$48,668)	A$94,506 (A$88815, A$98948)
OIT vs No treatment	A$60,840 (A$49,479, A$86,531)	A$888,976[a] (A$594,642, A$2,141,723)

Note: The 3.5-year time horizon is not appropriate for the primary economic evaluation, as it implicitly assumes that treatment benefits disappear immediately at the end of follow-up. The results nonetheless illustrate the importance of accounting for benefits that are expected to persist.

[a]OIT was estimated to be extremely costly in terms of QALY gains over the 3.5-year time horizon, as patient quality-of-life initially fell below baseline during the treatment period (Figure 1), resulting in a period of negative QALY gains before increasing.

© 2026 Huang L et al. *JAMA Network Open*.

eFigure 1. Remission and Quality of Life

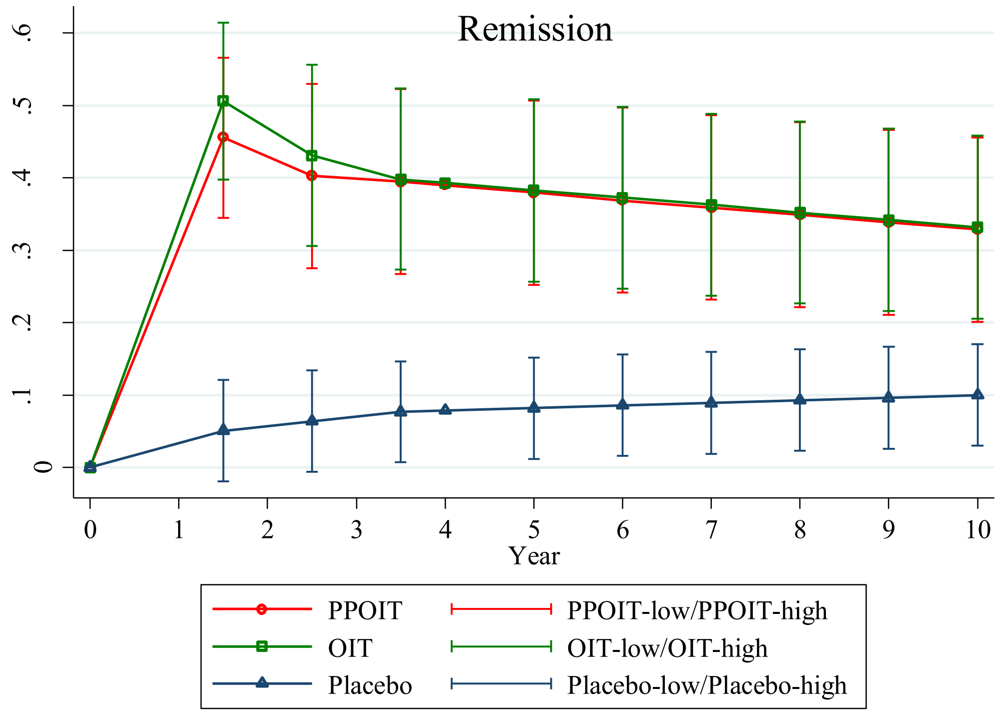

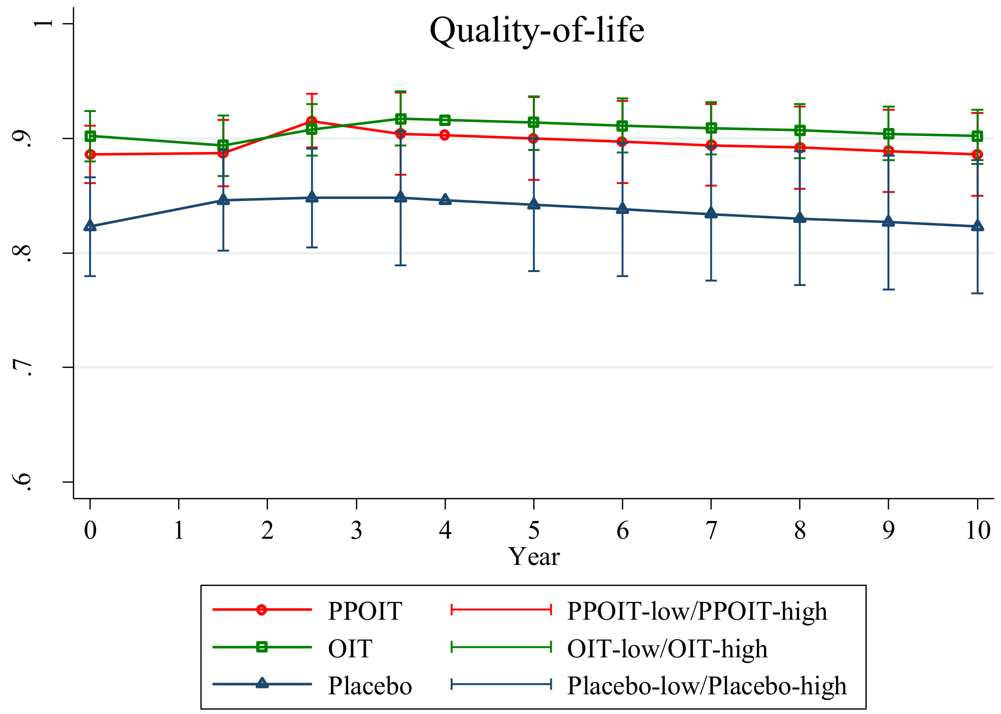

© 2026 Huang L et al. *JAMA Network Open*.

eFigure 2 Threshold Analysis for OIT Price Per Month (Australian $) at Which the Treatments Would Be Cost-Effective Given QALY Thresholds for Australia, the UK and the US

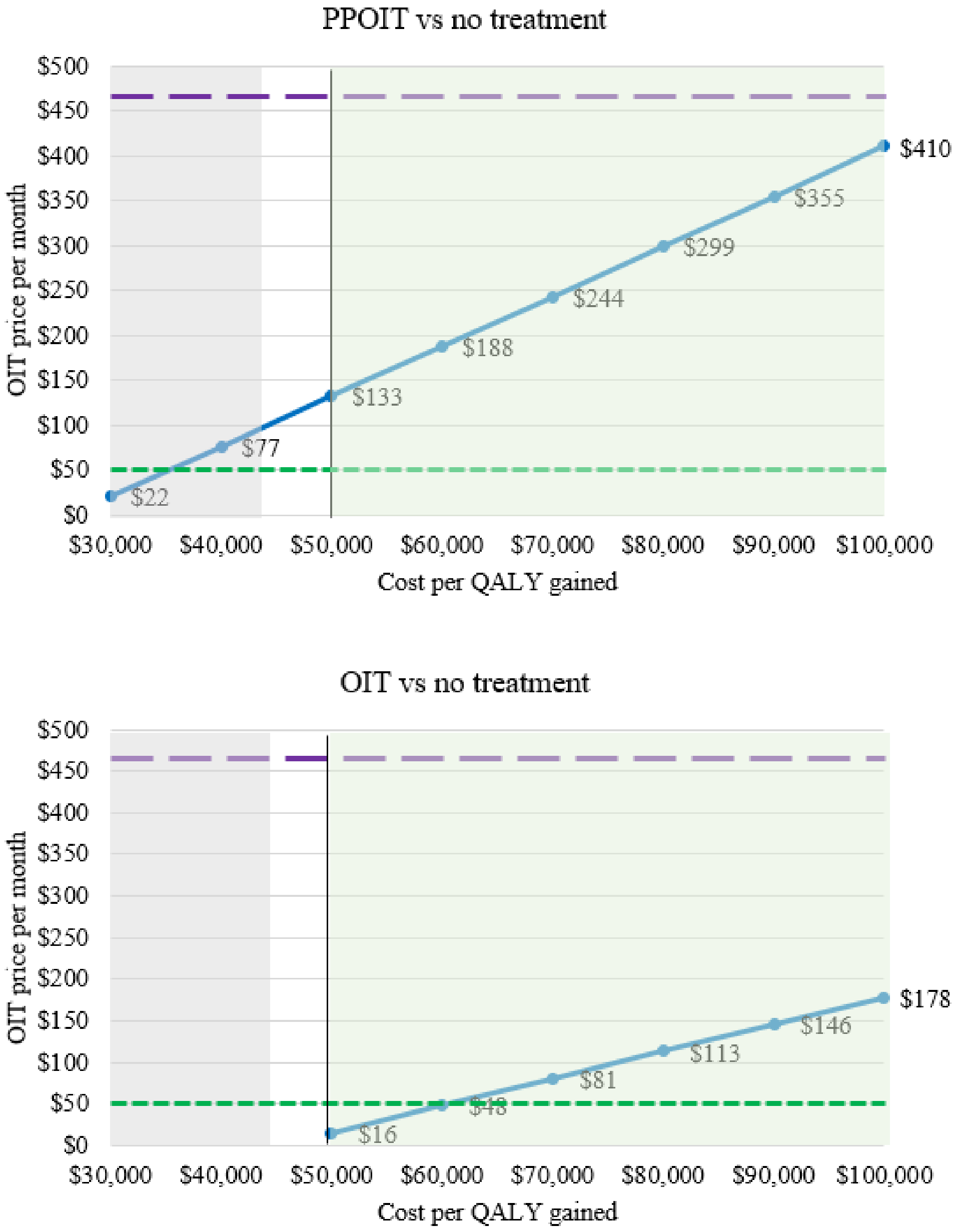

Trial estimated OIT price of $50 per month (green dash line ----)

NICE listed OIT price of $468 per month (purple dash line — –)

Australian QALY threshold (black vertical line |)

The UK QALY threshold (light grey area)

The US QALY threshold (light green area)

© 2026 Huang L et al. *JAMA Network Open*.
