# Supplementary material for: Cost-Effectiveness of Oral Immunotherapy Treatments vs No Treatment for Peanut Allergy in Children
Source: JAMA Netw Open. 2026 Mar 20;9(3):e262410. doi: 10.1001/jamanetworkopen.2026.2410 (PMC13005161; doi:10.1001/jamanetworkopen.2026.2410)
Supplement: Supplement 2. — Data Sharing Statement [file jamanetwopen-e262410-s002.pdf]

Data Sharing Statement

Huang. Cost-Effectiveness of Oral Immunotherapy Treatments vs no Treatment for Peanut Allergy in Children. *JAMA Netw Open*. Published March 20, 2026.
doi:10.1001/jamanetworkopen.2026.2410

Data
Data available: Yes
Data types: Deidentified participant data
How to access data: Requests can be sent to Prof Mimi Tang mimi.tang@rch.org.au.
When available: With publication

Supporting Documents
Document types: None

Additional Information
Who can access the data: Researchers whose proposed data use has been approved, with appropriate justification and a signed confidentiality agreement
Types of analyses: Research purpose only
Mechanisms of data availability: After approval with signed confidentiality agreement
